# Impact of H1N1 influenza on critical care and dependent services in Wales during winter 2010/2011

**DOI:** 10.1186/cc11084

**Published:** 2012-03-20

**Authors:** A Jones, D Hope, E Farley-Hill, J Parry-Jones

**Affiliations:** 1 Welsh Critical Care Networks, Newport, UK

## Introduction

Influenza H1N1 admissions to critical care from December 2010 to January 2011 had a significant impact on intensive care bed occupancy across Wales. Wales is relatively underprovided in critical care capacity and as a consequence the surge in admissions had a significant impact on both critical care and critical care dependent hospital services.

## Methods

Data were collected prospectively through the Critical Care Minimum Data Set: the number of critical care admissions with confirmed or highly suspected influenza, co-morbidities, mortality rate, level 3 bed day occupancy, number and mode of advanced respiratory support days, numbers of nonclinical and clinical transfers, and numbers of cancelled operations requiring critical care.

## Results

**Figure 1:**
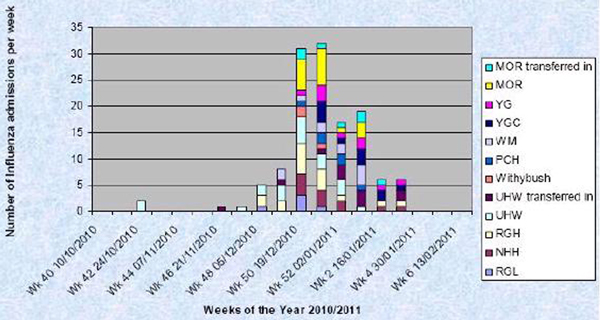
**Influenza admissions into critical care in winter 2010/2011**.

In a 10-week period 128 patients in Wales required critical care with influenza. A total of 1,692 level 3 bed days were required. There are 95 potential level 3 beds across Wales per day. Therefore >25% of level 3 beds over 10 weeks were occupied by influenza patients. Fifty percent of patients had significant comorbidities; pregnancy, COPD, morbid obesity, immunocompromise (Figure 1). The overall mortality rate for all affected critical care patients was 23.4%. Mortality was 25% in those with comorbidities and 22% in those without. The overall mortality rate for all affected patients treated in Wales during the 2009/10 influenza pandemic was 9.6%. The UK has fewer critical care beds per head of population than comparable nations, and Wales fewer still so critical care in Wales is more vulnerable to surges in admissions. This was apparent in the peak in nonclinical critical care transfers seen during this period, performed due to units exceeding their capacity, and in an increase in cancellations of elective surgery requiring critical care.

## Conclusion

The shortage of critical care capacity in Wales is made more apparent during times of increased critical care requirement such as the influenza in the winter 2010/2011. Hospital services are increasingly dependent on critical care, and government and health boards need to provide targeted increases in critical care bed provision to match those levels in other similar nations to mitigate the effect on critical care and dependent services due to surges in demand.

